# Enriching Eggs with Bioactive Compounds through the Inclusion of Grape Pomace in Laying Hens Diet: Effect on Internal and External Egg Quality Parameters

**DOI:** 10.3390/foods13101553

**Published:** 2024-05-16

**Authors:** Beatriz Herranz, Carlos Romero, Inés Sánchez-Román, Mónica López-Torres, Agustín Viveros, Ignacio Arija, María Dolores Álvarez, Sonia de Pascual-Teresa, Susana Chamorro

**Affiliations:** 1 Department of Food Technology, Faculty of Veterinary, Complutense University, Avda/Puerta de Hierro, s/n, 28040 Madrid, Spain; herranz@ucm.es; 2 Facultad de Ciencias y Artes, Universidad Católica Santa Teresa de Jesús de Ávila (UCAV), Calle Canteros, s/n, 05005 Ávila, Spain; carlos.romero@ucavila.es; 3 Animal Physiology Unit, Department of Genetics, Physiology and Microbiology, Faculty of Biological Sciences, Complutense University, c/José Antonio Novais 12, 28040 Madrid, Spain; inessa04@ucm.es (I.S.-R.); mltorres@bio.ucm.es (M.L.-T.); 4 Department of Animal Science, Faculty of Veterinary, Complutense University, Avda/Puerta de Hierro, s/n, 28040 Madrid, Spain; viverosa@vet.ucm.es (A.V.); arijai@ucm.es (I.A.); 5 Department of Characterization, Quality, and Safety, Institute of Food Science, Technology and Nutrition (ICTAN-CSIC), José Antonio Novais 6, 28040 Madrid, Spain; mayoyes@ictan.csic.es; 6 Department of Metabolism and Nutrition, Institute of Food Science, Technology and Nutrition (ICTAN-CSIC), José Antonio Novais 6, 28040 Madrid, Spain; s.depascualteresa@csic.es

**Keywords:** grape by-products, polyphenols, antioxidants, egg quality, gallic acid, vitamins, poultry, laying hen

## Abstract

(1) Background: Grapes and their associated by-products (such as grape pomace, GP) stand out for their polyphenol content, which makes them a source of bioactive compounds with antioxidant capacity. The aim of this research was to determine if the inclusion of 50 g/kg of GP in the diet of hens could enrich eggs with antioxidants and to study its effect on internal and external egg quality parameters. (2) Methods: A trial was conducted with two genetic lines of hens, which were fed either a control diet or a diet containing 50 g/kg of GP. Performance, internal and external egg quality, and egg yolk content of vitamins E and A and gallic acid were determined. (3) Results: In eggs laid by hens fed a GP diet, Haugh units and yolk color scores were enhanced, and eggshells became thinner, but without affecting the breaking strength. No dietary effect was observed on the vitamin contents of the yolk. A higher gallic acid content was observed in the yolks of eggs laid by hens fed the GP diet, suggesting that some dietary phenolic compounds could be transferred to the eggs. Hen genetics influenced egg weight, albumen Haugh units, shell thickness, and α- and γ-tocopherol concentration in yolks. (4) Conclusions: Dietary inclusion of GP improved the internal quality of eggs, enriching yolks with a phenolic compound but reducing shell thickness.

## 1. Introduction

The increase in food demand driven by population growth, coupled with the reduction in the supply of raw materials owing to factors such as climate change, poses a significant challenge for the agricultural and livestock sectors. Among the different processes associated with egg production, the feeding of hens represents the largest source of environmental impact [1]. Nonetheless, the revaluation of agrifood by-products through their incorporation into animal diets represents a promising strategy in order to reduce the environmental impact derived from waste generation and disposal, as well as a way to contribute to ensuring sustainable consumption and production patterns and reducing nutrient loss, as outlined in some of the Sustainable Development Goals (specifically, targets 2.4 and 12.5) of the 2030 Agenda [2]. Certain by-products have a high content of antioxidant compounds and could contribute to improving animal health and the quality of animal products. Among these, by-products originating from the winemaking process, such as grape pomace (GP), are a rich source of natural antioxidants like polyphenols. The GP consists of a mixture of stems, pulp, and seeds in varying proportions. Spain holds a significant position in the international wine economy, leading to the substantial availability of grape by-products, which have the potential to be used as ingredients in animal nutrition [3]. In recent years, some authors have demonstrated the antioxidant capacity of these by-products in poultry [4,5], their prebiotic effect [6], as well as their potential to improve meat quality [7,8]. Furthermore, the identification of phenolic metabolites in the intestinal content and plasma of chickens that had consumed grape by-products [9,10] suggests that these compounds could be transferred to tissues where they may exert their antioxidant effect. Although less research has been conducted with laying hens, it has also been shown [11,12,13] that the inclusion of grape by-products in the diet of hens can modify egg composition (e.g., yolk fatty acid profile by reducing the proportion of saturated fatty acids while increasing that of polyunsaturated ones) and can improve the oxidative stability of yolk lipids during refrigerated storage. Moreover, recent research has demonstrated that it is possible to reduce oxidation and increase egg fertility by incorporating grape polyphenols into the diet of hens [14,15]. Additionally, phenolic metabolites have been identified in the plasma of hens after feeding the latter with diets containing grape seed extract [16]. These results suggest that certain grape polyphenols could also transfer to the egg and exert their antioxidant action there, as reported by Vlaicu et al. [12] in laying hens fed a diet containing grape seed. However, there is no information demonstrating which phenolic metabolites originating from dietary grape polyphenols can be deposited in the egg by laying hens and, therefore, whether it is possible to enrich eggs with these antioxidant compounds. Due to the efficiency of laying hens in depositing nutrients from the diet into the egg, incorporating bioactive compounds into laying hens’ diets could be a successful strategy to enhance the nutraceutical value of eggs. Actually, it has been proven that when antioxidants are included in the feed of laying hens, the metabolism of hens allocates these dietary antioxidants preferentially in the egg instead of in the body [17].

On the other hand, several authors have highlighted the ability of dietary grape polyphenols to increase the content of vitamin E in plasma and tissues in birds [5,7,8,10,18]. Polyphenols, similarly to vitamin C, can interact with radicals generated during the metabolism of vitamin E, thereby recycling alpha-tocopherol in plasma and tissues. Furthermore, it has been demonstrated that it is possible to increase the content of vitamin E in eggs by means of hens’ diet [19,20], making it interesting to study whether the dietary inclusion of GP would also allow an increase in the deposition of vitamin E in egg yolk. Likewise, considering the potential interaction in intestinal absorption between fat-soluble vitamins [21], modifications in the deposition of vitamin E could affect the content of vitamin A in egg yolk.

Previous research also conducted with laying hens [11] has proven that a dietary dose of 60 g/kg of GP enabled several improvements in egg quality (e.g., a higher proportion of polyunsaturated fatty acids in the yolk and fewer oxidation of yolk lipids) that could not be achieved with a dose of 30 g/kg of GP, but at the same time, the diet including GP at 60 g/kg showed some drawbacks like impaired feed conversion ratio and reduced protein digestibility. Hence, it seems pertinent to evaluate whether an intermediate dietary dose of GP could also improve egg quality without affecting the negatively productive performance of laying hens.

Thus, the aim of this study was to determine if the inclusion of 50 g/kg of GP in hens’ diet might produce enrichment in phenolic compounds in the egg and to study the effect of this dietary inclusion on internal and external egg quality parameters and on α- and γ-tocopherol and retinol contents in egg yolk. 

## 2. Materials and Methods

### 2.1. Standards and the Tested Product

All chemicals used were HPLC analytical grade, and the water was ultrapure. Gallic acid, catechin, cyanidin 3-O-glucoside, quercetin 3-O-glucoside, α-tocopherol, γ-tocopherol, and retinol were purchased from Sigma Chemical Co. (St. Louis, MO, USA). Methanol, ethanol, and hexane were obtained from Panreac (Castellar del Vallés, Barcelona, Spain).

The grape by-product (whole pomace), consisting of a mixture of seeds and skins, was obtained fresh immediately after racking (following 15 days of fermentation) and mechanical separation of stems. Grapes originated from the September 2021 harvest season and were obtained at Roquesán Cooperative Winery (Quemada, Burgos, Spain). The GP was dried in an oven at 50 °C for 4 days and subsequently stored in darkness. Thereafter, GP was ground with a 1-mm screen and incorporated into the experimental diet. The nutritional composition and the phenolic profile of GP are reported in Table 1 and Table 2, respectively.

### 2.2. Birds and Diets

This trial was conducted at the experimental facilities of the Faculty of Veterinary Science of the Complutense University of Madrid (Spain). Thirty ISA white and thirty ISA brown laying hens were used. When the experiment began, all the hens were 22 weeks old. Hens of each genetic line were allocated randomly to six wire cages (five hens per cage) under controlled environmental conditions in a completely enclosed fan-ventilated building with a daily light program of 16 h (light was switched on at 06:00 am). The cages used in this research work were enriched cages meeting the requirements set by Council Directive 1999/74/EC (cages with an area of 750 cm^2^ per hen, with a nest, and with at least 0.20 m of height at any point within the cage, 0.15 m of perch per hen, and 0.12 m of feeder length per hen). Research procedures were approved by the Complutense University of Madrid Animal Care and Ethics Committee (protocol code NP0926052022-2022) in compliance with the principles for the Care and Use of Animals for Scientific Purposes of the Ministry of Agriculture, Fishery, and Food.

Hens of each genetic line were fed either a control diet or a diet including GP at 50 g/kg (3 replicate cages of each genetic line per diet). The ingredients and nutrient composition of the two experimental diets are shown in Table 3. Diets were formulated to be isocaloric, isonitrogenous, and isofibrous and to contain the same amount of calcium and phosphorus. To make diets isofibrous, straw was included in the control diet. Diets were exempt from colorants and antioxidants. Feed, offered in mash form, and water were provided ad libitum throughout the whole experiment.

Thus, the present trial considered the following four treatments arranged in a factorial 2 × 2 design: (1) ISA white hens—control diet; (2) ISA white hens—GP 50 g/kg diet; (3) ISA brown hens—control diet; (4) ISA brown hens—GP 50 g/kg diet.

### 2.3. Hen Performance Assessment and Egg Collection

After a two-week adaptation period, laying hen performance was assessed for four weeks (from week 24 to week 28 of age). Feed intake by laying hens was recorded weekly and then divided by the number of birds per cage and by seven in order to determine the daily feed intake per hen. All laid eggs were collected daily, and the number of eggs obtained in each cage was written down. All eggs were then weighed. Daily egg mass and feed conversion ratio per cage were calculated according to the following formulas:

Daily egg mass = (Daily egg production/100) × Average egg weight
(1)

Feed conversion ratio = Feed intake/Daily egg mass
(2)

On the first and second days of week four of the trial (week 27 of the age of hens), 9 freshly laid eggs were collected daily per treatment (daily, 3 eggs per replicate of each of the four treatments), individually weighed, and used for determination of shell proportion, shell thickness, albumen Haugh units, and egg yolk color.

On the third and fourth days of week four of the trial (week 27 of the age of the hens), 12 freshly laid eggs were collected daily per treatment (daily, 4 eggs per replicate of each of the four treatments). These eggs were broken, and for each one of them, the yolk was separated from the egg white. The 24 yolks thereby obtained per treatment were frozen at −20 °C, freeze-dried, and subsequently used for the determination of α- and γ-tocopherol and retinol content.

Once the hens reached the age of 28 weeks, the trial was continued for another six weeks only with the ISA white laying hens. These 30 hens kept on eating the same diet they had been receiving since they were 22 weeks old (15 ISA white laying hens per diet). On weeks 31, 32, and 33 of the age of the laying hens, all eggs produced were collected daily, counted, marked, and weighed. From the eggs obtained in weeks 31 and 32, 36 eggs per cage were set aside. Nine out of these 36 eggs were used on collection day for assessment of albumen Haugh units, yolk color score, and eggshell quality traits (shell thickness, shell-breaking strength, total rupture area, and shell rupture force peaks). The remaining 27 eggs per cage were stored in a refrigeration chamber at 4 °C. At 15, 21, and 31 days of storage, 9 eggs per cage were taken out of the chamber and also used for the assessment of albumen Haugh units and yolk color score. On week 32 of the age of the laying hens, 9 additional eggs per cage were used for measurement of shell thickness.

On the first day of week 33, four freshly laid eggs were taken per cage and broken. For each one of these eggs, the yolk was separated from the albumen. The four yolks originating from the same cage were pooled, hence obtaining three pools per dietary treatment. These six pools of yolks were frozen, freeze-dried, and later used for the determination of gallic acid concentration.

### 2.4. Measurements of Egg Quality Parameters

Eggshells were washed, the testaceous membranes were removed, and then the shells were dried in the air at room temperature for 48 h. Shell thickness was assessed at the egg equator with a digital Mitutoyo shell thickness micrometer (Kawasaki, Japan). 

Albumen height was measured with a QCH device (TSS, York, UK). Haugh units were determined thereafter with the formula:

Haugh units = 100 × log (h − 1.7 × w^0.37^ + 7.57) (3)

where h = albumen height (mm) and w = egg weight (g) [22].

At week 27 of the age of laying hens, the color of egg yolk was measured using a Minolta Chromameter (Model CR-400, Minolta Co., Osaka, Japan). According to the CIELAB color space, color values were expressed as L* (lightness), a* (redness), and b* (yellowness). The white standard was used to calibrate the chromameter. In eggs laid by hens aged 31 and 32 weeks, yolk color was assessed using the Roche Yolk Color Fan, and data were expressed in the standard DSM Roche Fan values (from 1 for light yellow to 15 for orange). 

Mechanical measurements in the shells were performed using a TA-XT2i Texture Analyzer (Stable Micro Systems, Ltd., Godalming, UK) provided with Texture Exponent software (version 6.1.20.0) and equipped with a 30 kg load cell. A compression test was performed with a 45 mm diameter flat cylindrical stainless-steel probe (SMS P/45). Eggs were placed on a specific stationary platform in a horizontal position relative to the probe. This specific platform has three vertices and a central hole designed for the shape of the egg, collecting the white and the yolk separately. A pressure force was applied with the probe until the eggshell was completely broken. The test speed was set at 1 mm/s, considering a trigger test force of 0.001 N. Data acquisition was performed at a rate of 500 pps (points per second). From the force–time curves, the shell-breaking strength (N) and the total rupture area (N.s.), corresponding, respectively, to the maximum force and area required to crush the eggshell surface, were obtained. In addition, the number of shell rupture force peaks, calculated for a drop in force higher than 0.05 N, was derived.

### 2.5. Chemical Analyses

Dry matter (930.15), crude protein (976.05), crude fiber (978.10), ashes (942.05), and ether extract by Soxhlet fat analysis after acid hydrolysis (method 920.39) were analyzed in accordance with the methods of the Association of Official Analytical Chemists [23]. Chemical analyses were conducted in triplicate.

#### 2.5.1. Vitamins A and E Determinations

Retinol and α- and γ-tocopherol extractions were performed following the protocol indicated by Claeys et al. [24]. In an amber glass tube with a screw cap, 200 mg of egg yolk were weighed and stirred vigorously with 6 mL of ethanol with ascorbic acid (5 g/L). It was incubated in a water bath at 78 °C for 30 min, mixing the contents with a glass rod after 10 min of incubation. The tube was cooled under tap water, 1 mL of distilled water was added, and it was incubated in an ice bath for 15 min. It was centrifuged for 5 min at 1100× *g*, and the supernatant was transferred to another amber tube. Two milliliters of KCl (57.5 g/L) and 2 mL of hexane (containing 0.02 g/L BHT) were added, vigorously mixed, and centrifuged for 5 min at 720× *g*. The hexane phase was transferred to another amber tube using a Pasteur pipette. The hexane extraction step was repeated, and the pooled hexane phase was evaporated under N_2_ gas. Finally, the precipitate was dissolved in 1 mL methanol, filtered through a 0.2 μm cellulose membrane (Scharlab S.L.), and collected in an amber vial. Simultaneous determination of vitamins A and E was performed by HPLC following the methodology previously described by Granado-Lorencio et al. [25].

A reversed-phase HPLC technique was used, with a Gilson 305 pump and a 5 μm (100 mm × 4.6 mm) KromaPhase C18 chromatography column. The mobile phase was methanol–water (97:3) with the following conditions: 1 mL/min flow rate, around 0.78 kpsi pressure, and 25 °C. A Jasco UV-2075Plus UV detector was used, and absorbance was recorded at 292 nm for α- and γ-tocopherol and 325 nm for retinol. Quantification was carried out immediately after extraction. Standard solutions of known concentrations of α-tocopherol, γ-tocopherol, and retinol (Sigma-Aldrich^®^, St. Louis, MO, USA) were injected, and peak areas were used to calculate α-tocopherol, γ-tocopherol, and retinol concentrations in the samples.

#### 2.5.2. Phenolic Compounds

For the extraction of phenolic compounds in GP and diets, 50 mg of the sample was placed in a capped centrifuge tube and suspended in 1 mL of acidified methanol 50:50 vortexed and sonicated over 15 min. Thereafter, samples were centrifuged at 10,400× *g* for 10 min at 4 °C, the supernatant was collected, and the resulting pellet was re-suspended in 500 μL of methanol–water (50:50) acidified with formic acid (0.1%), with the extraction process being repeated twice. The obtained supernatants were combined (2 mL in total), filtered (0.45 µm), placed in vials, and used to quantify the total extractable polyphenols by Folin–Ciocalteu method and for subsequent HPLC–QTOF-MS phenolic compound identification. 

For egg yolk extraction, 200 mg of freeze-dried egg yolk were re-suspended in 1.5 mL of acidified methanol–water (80:20, 0.1% formic acid), and taxifolin was added as an internal standard. Samples were gently mixed for 1 h, sonicated over 15 min, and centrifuged at 10,400× *g* for 10 min at 4 °C. The supernatant was collected, and the resulting pellet was extracted, repeating the process twice. Then, supernatants were mixed, concentrated (N atmosphere), re-suspended in 500 μL of methanol–water (50:50, 0.1% formic acid), and placed in vials for subsequent HPLC–QTOF-MS analysis of phenolic compounds.

The identification of the different compounds present in GP and egg yolk was performed using HPLC coupled with a mass spectrometer (HPLC-QTOF-MS) following the prevailing method for the analysis of polyphenols [26]. The HPLC (Agilent 1200, Agilent Technologies, Waldrom, Germany) has a quaternary pump (model G1311A) coupled with a diode array detector (Agilent model G1315B) and an Agilent 6530 Accurate-Mass QTOF-MS with Electrospray Ionization (ESI) with Jet Stream technology (Agilent Technologies, Santa Clara, CA, USA). Separation was performed on a Phenomenex Luna C18 column (5 μm, 4.6 mm × 150 mm; Phenomenex, Alcobendas, Spain), set thermostatically at 25 °C. A gradient between solvent A (water/formic acid, 99.9:0.1, *v*/*v*) and solvent B (acetonitrile/formic acid, 99.9:0.1, *v*/*v*) was applied at a flow rate of 0.5 mL/min as follows: 10% B at 0 min, 30% B at 30 min, 35% B at 35 min, 40% B at 45 min, 10% B at 50 min, and 10% at 60 min. The electrospray ionization (ESI) parameters were as follows: drying gas flow, 8 L/min; nebulizer pressure, 45 psi; gas drying temperature, 325 °C; sheath gas temperature, 300 °C; sheath gas flow, 11 L/min; capillary voltage, 4000 kV; and fragmentator, 120 V. The ESI was operated in a positive and negative mode to provide extra certainty in the determination of the molecular masses. Compounds were identified using extracted-ion chromatogram (EIC) data by comparing with external standards and confirmed by tandem mass spectrometry fragmentation spectra (MS/MS). For mass spectrometry experiments, 20 V collision energy was used. Spectral signal data were additionally acquired at 280, 320, and 520 nm. Data acquisition and processing were performed with Masshunter Data Acquisition B.05.01 and Masshunter Qualitative Analysis B.07.00 SP2 software (both from Agilent Technologies, Santa Clara, CA, USA). Compounds were identified by comparing mass spectra and retention time with the corresponding standard, if available. The quantification was performed by interpolation into the calibration curve of the standard or structurally related compound used to quantify (equivalent) and expressed as g per g of dry matter (DM) as follows: epicatechin for oligomeric procyanidins, quercetin-3-O-glucoside for flavonols, and cyanidin-3-O-glucoside for anthocyanidins.

### 2.6. Statistical Analysis

Data from variables were subjected to a one-way analysis of variance (ANOVA) with laying hen genetics, diet, and their interaction as the main sources of variation by using the general linear model procedure (Version 9.4, SAS Institute Inc., Cary, NC, USA). When the effect was found to be significant (*p* < 0.05), all the treatment means were compared using a *t*-test. 

The cage with five hens represented the experimental unit for performance variables, whereas the egg was the experimental unit for external and internal quality parameters and for α- and γ-tocopherol and retinol concentrations. A pool of four egg yolks from the same dietary treatment constituted the experimental unit for gallic acid concentration.

## 3. Results and Discussion

### 3.1. Phenolic Compounds in Grape Pomace

As can be seen in Table 2, the composition of the grape pomace used to feed the hens could be considered especially rich in anthocyanins, which accounted for nearly 70% of total polyphenols, with malvidin-3-glucoside being the most abundant anthocyanin (1.61 mg/g). Flavanols, on the other hand, represented 20% of the total phenolic compounds, being, in this case, the monomers catechin and epicatechin, the most abundant flavanols. Furthermore, it could be highlighted that delphinidin-3-glucoside and free quercetin were present at high concentrations (0.25 and 0.09 mg/g, respectively). In general, the composition of the grape pomace used in the current trial is in agreement with previously published works also dealing with grape pomace [27]. 

### 3.2. Productive Performance of Laying Hens

The genetics of laying hens and the diet consumed are the main sources of variation affecting egg production and quality [28,29]. Conventionally, two types of laying hens are used worldwide in laying hen husbandry: light hens (hens laying white eggs) and semi-heavy hens (hens laying brown eggs). Hence, in the present study, a strain of light hens (ISA white) and a strain of semi-heavy hens (ISA brown) were used and compared.

Table 4 shows the effect of hen genetics and dietary treatment on the productive performance of hens in the present research work. In keeping with productive results published in ISA Poultry performance guides, daily egg mass was lower for ISA white than for ISA brown hens (55.2 vs. 58.0 g/d, *p* = 0.004). However, since the feed intake of ISA white hens was also lower than that of ISA brown hens (111 vs. 123 g/d, *p* < 0.001), ISA white hens turned out to be more efficient in egg production (lower feed conversion ratio, 2.02 vs. 2.13, *p* = 0.012). Both strains of hens showed very high daily egg production (97.1%, on average), as should have been expected for selected hens of this age (at 24–26 weeks of age, these laying hens reach peak egg production). In the current research work, the dietary inclusion of GP at 50 g/kg had no effect on egg weight, nor did the dietary inclusion of grape seed at 30 g/kg affect egg weight in the study of Vlaicu et al. [12]. On the contrary, in a previous study [11], the inclusion of GP at 30 or 60 g/kg in the diet of laying hens reduced the weight of eggs laid. The different effects observed between these works could be attributed to the different grape polyphenol concentrations in the experimental diets. Even if polyphenols have long been considered antinutritional factors hindering protein digestibility, feed conversion ratio, which is a parameter largely used in animal science to assess the efficiency of livestock, has been reported to remain unaffected [12,14], just like it happened in the current research work, or even to be improved [11,13] in laying hens when including grape by-products (either grape seed or grape pomace) in the diet.

### 3.3. Egg Internal Quality

The effect of genetics and diet on various parameters determining the internal quality of freshly laid eggs is shown in Table 5. The Haugh units (HU) are an important indicator used to assess albumen quality based on variables such as egg white height and egg weight [30]. In this context, a higher value of HU is associated with better protein quality in the egg, in accordance with the height of the albumen at its point of maximum viscosity and thickness. Indeed, high values of HU are largely considered indicators of egg freshness [31]. As can be observed in Table 5, both strains of hens exhibited high HU values and, consequently, excellent quality of albumen, with no significant differences due to dietary inclusion of GP being detected. These results are in disagreement with those obtained by Romero et al. [11], who reported an increase in the HU of light-laying hens (Hy-Line strain) fed diets including GP (either at 30 or 60 g/kg) as compared with the control treatment. Nevertheless, it should be noted that the HU values of the present work are 22.5% higher, on average, than those of Romero et al. [11], and hence, it could be surmised that, since the HU values obtained in the current study in hens fed the control diet were already very high, very little further improvement could be achieved with the dietary inclusion of GP. Other authors, such as Zhu et al. [31], found a decrease in HU values in commercial Lohmann pink-shell laying hens when feeding them with different tea polyphenols in comparison with the control group. These differences could be explained by the different nature and content of the polyphenols present in tea and GP. Additionally, the use of different breeds could also contribute to explaining the different responses. 

Concerning the yolk color, when determined according to the CIELAB color space, results (Table 5) showed that lightness (L*) was not affected either by diet or hen genetics. However, the parameter a* (redness tendency) was affected by both treatments, with a significant decrease in a* observed in eggs from hens that had consumed GP (2.73 vs. 3.53, *p* < 0.001) and higher values being found in semi-heavy hens (3.33 vs. 2.93, *p* = 0.008). Likewise, the parameter b* (yellowness tendency) tended (*p* = 0.07) to decrease (25.9 vs. 27.5) when the diet of hens included GP. Therefore, it seemed that eggs from hens fed the diet, including GP, exhibited rather orange-colored yolks, which may be more attractive from a sensory perspective for consumers [32].

For none of the internal quality parameters assessed in freshly laid eggs, the interaction between hen genetics and the dietary inclusion of GP was found to be significant. Hence, the effect of diet and storage time (15, 21, and 31 days) of eggs on HU and yolk color score, now measured using the Roche scale, was only evaluated with one strain of hens (light-laying hens). These results are provided in Table 6. In keeping with the decreasing effect on HU due to storage duration observed by Grashorn et al. [33], a significant reduction (*p* < 0.001) over time of HU was detected in the present study (96.7, 93.4, 87.4, and 69.6 at 0, 15, 21, and 31 days of storage, respectively). On the contrary, the dietary inclusion of GP tended (*p* = 0.06) to increase the HU (87.8 vs. 85.8), with the interaction between diet and storage time not being significant. The positive effect on HU resulting from the dietary inclusion of GP had not been previously detected when hens were 24 to 28 weeks old. Nonetheless, at that age, the diet containing GP led to HU values 1.45% higher than those of the control group, but this difference did not reach a significance level. Now, in hens aged 31 to 34 weeks, the effect of dietary GP could have become significant because a higher number of eggs per treatment was evaluated. Similarly, dietary inclusion of GP (either at 30 or 60 g/kg) also increased egg HU in a previous study [11]. As aforementioned, HU is related to the quality of albumen. The changes that this portion of the egg undergoes during storage have been well studied for decades. Reduced HU values observed throughout storage indicate a decline in the functional properties of albumen. The consistency of the albumen was compromised when the HU score fell below 70 during the storage period [34]. Shan et al. [35] concluded that the highly glycosylated ovomucin in the egg white undergoes degradation as its glycan chain hydrolyzes, accompanied by a thinning of the egg white. Another study [36] has suggested that ovalbumin gradually converts to S-ovalbumin during storage, with the content of S-ovalbumin being negatively correlated with HU. 

As regards yolk color, assessed according to the Roche scale, no effect due to the storage time of eggs was detected (Table 6), whereas the presence of GP in the diet at 50 g/kg increased (*p* < 0.001) the yolk color score by 9.36%. Again, as previously observed, from 24 to 28 weeks of age, the diet, including GP, made egg yolks look more orange. In previous research [11,12], the dietary inclusion of GP at 60 g/kg or that of grape seed at 30 g/kg increased the yolk color score by 12.8% and 8.35%, respectively. While this index does not imply nutritional value, it is crucial for consumer acceptance of eggs, and it has been largely proven that egg yolk color can be influenced by the diet fed to the hens [37,38]. Actually, it has been observed that European consumers prefer yolk coloration between 9 and 14, with differences between northern and southern countries. Southern countries tend to prefer yolks with more intense colors (11–14), while northern countries prefer paler yolks (9–10) [39]. In this study, means lower than 11 (ranging from 9.29 to 10.7) were obtained, which meets the quality standards of only northern European countries. This could be explained by the absence of colorants in both feeds. In a previous study [40] aimed at finding natural pigments to replace synthetic ones in the diets of laying hens, it was concluded that, among the studied ingredients (dandelion, marigold, and basil), only eggs from diets supplemented with marigold flowers, a compound with a high content of xanthophylls, showed acceptable yolk color values for southern European countries. Tufarelli et al. [41] also indicated that hens consuming 150 g/kg of dehydrated tomato pomace and 50 g/kg of dehydrated GP showed significantly higher yolk color than those fed a diet containing flaxseed meal. In the current research, the interaction between diet and storage time was not found to be significant, but Grčević et al. [42] reported that, while storage duration did not affect the yolk color value of control eggs, eggs from experimental diets supplemented with lutein (in the form of powdered marigold extract) showed significantly higher yolk color values in eggs stored for 20 days.

### 3.4. Egg External Quality

The external quality of eggs was evaluated on the basis of shell thickness and mechanical parameters derived from force–time curves (shell-breaking strength, total rupture area, and shell rupture force peaks). When hens were aged 24 to 28 weeks, no effect of dietary inclusion of GP was observed on shell thickness (Table 5), whereas in 31- to 34-week-old laying hens (Table 7), the diet containing GP resulted in an impairment of shell thickness (361 vs. 424 μm, *p* < 0.001). Polyphenols are known to have the ability to chelate calcium ions [43], which could have reduced the intestinal absorption of calcium in hens fed the GP diet and, thereby, hindered the correct formation of shells in the uterus. This negative effect of the dietary inclusion of flavonoids on the shell thickness of eggs was also observed by Zhu et al. [31]. These authors showed that the addition of six tea by-products with different caffeine contents to the diet of laying hens caused a marked reduction in shell thickness and weight. They reported that high quantities of caffeine could eventually bring about shell fractures caused by calcium loss and availability. Finally, as commonly observed in laying hen husbandry [29], eggshells were thinner for light hens than for semi-heavy ones (370 vs. 386 μm, *p* = 0.038). In this sense, Silversides and Scott [44] found that the shell percentage in the egg was lower for ISA white hens than for ISA brown hens. Actually, differences between strains or breeds in the characteristics of eggshells have already been highlighted in previous studies [45,46]. Nevertheless, the physiological processes behind these differences due to bird genetics remain unclear. Recent research has suggested that genetic variation in the quality traits of eggshells may be related to different eggshell biomineralization [47,48].

**Figure 1:**
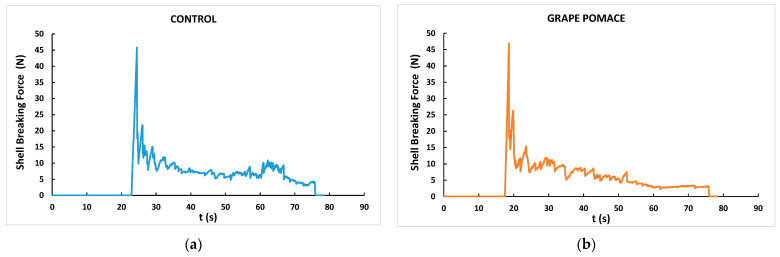
Examples of shell-breaking force for eggs from ISA white laying hens fed control (**a**) and grape pomace (50 g/kg) (**b**) diets.

Figure 1 is an example of the shell-breaking force–time curves of samples from the control (Figure 1a) and GP diet (Figure 1b) groups. Eggs were compressed in their equatorial position until they were completely broken, obtaining very similar force–time curves. In eggs from both dietary treatments, a noticeable maximum peak of force was observed (nonetheless, after 20 s for the control birds but before 20 s in eggs from hens fed the GP diet), corresponding with the shell-breaking strength, followed by numerous smaller fracturability force peaks until the total rupture of the eggs ends, which happened at around 80 s. The mechanical parameters derived from these force–time curves are also shown in Table 7. Unlike what was detected for shell thickness, no negative effect of dietary inclusion of GP was found on any of the mechanical parameters, for which no significant differences were observed between the dietary treatments. Likewise, Vlaicu et al. [12] reported that the dietary inclusion of grape seed at 30 g/kg in the feed of laying hens neither influenced the shell-breaking strength.

On the other hand, it is important to indicate that the number of eggs per treatment in which the shell-breaking test was carried out was quite lower (*n* = 27) than that of eggs used to determine shell thickness (*n* = 54). This was due to the fact that textural parameters were assessed with a tetrameter, which is instrumental equipment with a high degree of precision, accuracy, and reproducibility, making it possible to obtain precise and reproducible results even with small numbers of samples. Nonetheless, it is also true that it would be recommendable to increase the number of samples in future studies and, thereby, confirm that this reduction in shell thickness does not have a negative effect on shell strength.

### 3.5. Bioactive Compounds Measurements

#### 3.5.1. Vitamins A and E Determinations

The effect of diet and genetics on the composition of vitamin E (α- and γ-tocopherol) as well as vitamin A (retinol) in egg yolk is presented in Table 8. No effect on vitamin E and A concentrations was observed when diets were compared. Previous research in chickens has shown an increase in α- and γ-tocopherol concentrations in plasma and meat after feeding chickens a diet including GP [7,8]. As far as seen in the literature, this is the first time in which the amount of vitamins A and E is studied in the egg yolk after the inclusion of GP in the diet of laying hens. The increase in vitamin E in plasma obtained by Romero et al. [7] may not be translated into the egg yolk, as in our case, due to the regulatory role of the liver in the incorporation of certain products into the egg yolk. Thus, the liver may need to be saturated before certain substances are incorporated into the egg yolk, and this could be the case with the studied vitamins [49]. A study performed in laying hens with a hempseed-supplemented diet observed an increase in α- and γ-tocopherol in egg yolks [20], but there are some important methodological differences (duration of the treatment, strains raised, degree of supplementation, etc.) between their study and ours that could account for the absence of the effect of the diet in our research work. It is also noteworthy to mention that our GP diet contained a higher percentage of sunflower oil than the control diet, in order to balance the energetic input between diets. Sunflower oil is rich in polyunsaturated fatty acids that may make the eggs more susceptible to oxidation [50]. Despite this, we have not found lower levels of α- and γ-tocopherol in the egg yolks of hens fed with the GP diet, which may indeed indicate a positive effect of dietary GP on the amount of vitamin E in the egg yolk. We hypothesize that polyphenols present in GP could stimulate the turnover from α-tocopheroxil to α-tocopherol, reducing the last one that is oxidized under oxidative stress and contributing to regenerating the reduced α-tocopherol pool. Another explanation could be that the polyphenols get oxidized, preventing the antioxidants present in the egg yolk from being consumed. Actually, it has been proven in previous research that diets containing grape polyphenols result in reduced peroxidation of yolk lipids [11,13,51]. The concentration of retinol in egg yolks was not different in hens fed the GP diet than in hens fed the control diet. The amount of retinol in eggs seems to be closely related to its presence in the diet, either in the form of retinol or its precursors, carotenoids [52,53]. In spite of the high number of carotenoids present in the grapes, these are usually degraded during the alcoholic fermentation [54], and we believe that the amount of carotenoids remaining in the GP incorporated into the diet may not have been enough to increase retinol content in the egg. 

Regarding the influence of genetics on the amount of vitamins A and E in egg yolks, we observed a higher amount of α-tocopherol in ISA brown egg yolks than in eggs laid by ISA white hens. However, the opposite was observed when γ-tocopherol was analyzed, being higher in ISA white egg yolks than in ISA brown. Differences between the strains used have also been shown in other parameters of this study, indicating the importance of genetics on egg quality. In agreement with this, other studies have also described the effect of genetics on the amount of vitamins [53] and quality parameters [55]. In line with our results, a higher concentration of α-tocopherol was found in eggs laid by Rhode Island hens, which are semi-heavy hens just like the ISA brown hens of the present study, in comparison with the eggs of Leghorn hens, light hens, and those of the ISA white strain [53]. When analyzing retinol concentration, we found no influence of genetics on the amount of this vitamin in the egg yolk. Remarkably, as shown in Table 6, we did not find any interaction between diet and genetics, indicating hence that the lack of effect of GP diet was consistent independently of genetics.

#### 3.5.2. Phenolic Compounds

**Figure 2:**
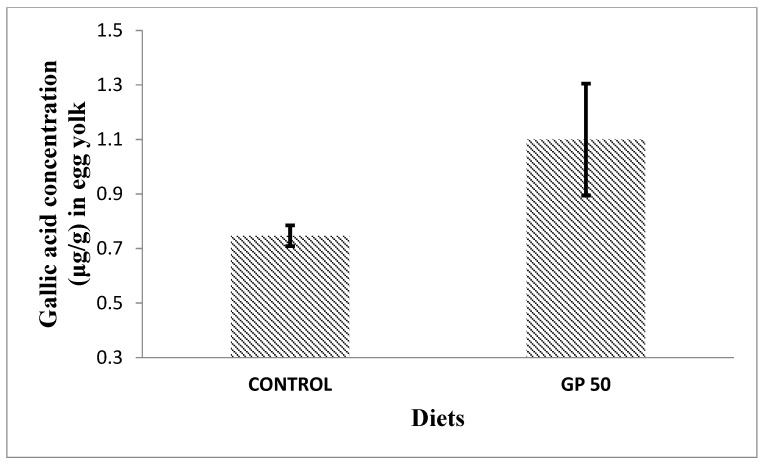
Effect of dietary inclusion of grape pomace (GP) on yolk gallic acid concentration (μg/g) in eggs of 31- to 34-week-old ISA white laying hens. Data are means of three replicates per treatment (a pool of four egg yolks per replicate). Figure shows mean ± standard deviation. SEM = 0.104, *p* = 0.074.

**Figure 3:**
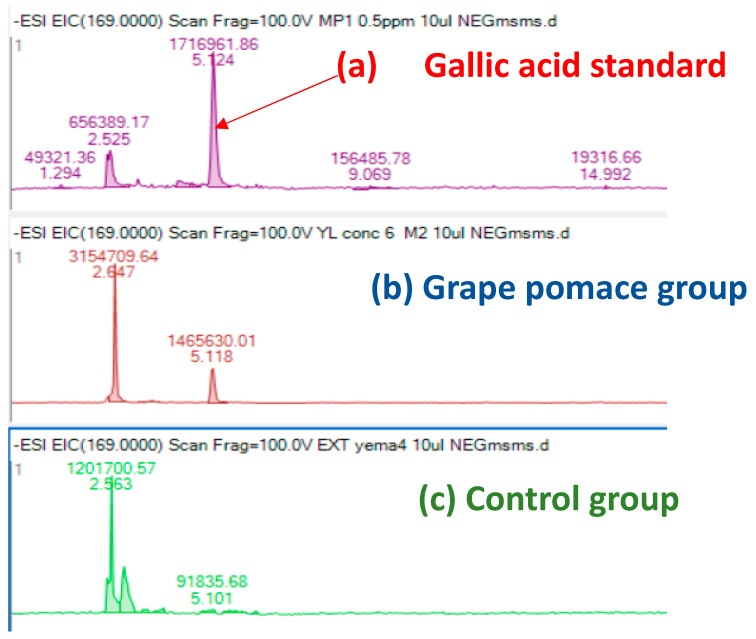
Extracted ion chromatogram (EIC) of gallic acid standard (**a**) and gallic acid content in egg yolk from hens fed a diet containing 50 g/kg of grape pomace (**b**) or a control diet (**c**).

Laying hens have the capacity to transfer health-promoting components, such as vitamins and minerals, from the diet into eggs. Thus, incorporating bioactive compounds into laying hens’ diets could be a successful strategy to enhance the nutraceutical value of eggs. Among them, α-tocopherol, the most common antioxidant utilized in animal nutrition, is easily deposited in egg yolks from the diet [20,56]. Additionally, eggs can be enriched with other antioxidants such as carotenoids (mainly xanthophylls) or selenium [56,57]. Regarding the transfer of polyphenols into eggs, a few studies have been carried out, in which the transference of phenolic compounds present in soybean [58] and corn/wheat [59] was studied. Saitoh et al. [58,60] studied the biotransformation of soy isoflavone-glycosides in laying hens and detected preferential accumulation of equol, a metabolite of daidzein, into the egg yolk. However, so far, no further studies have been conducted regarding the transfer of phenolic compounds/metabolites into the egg. On the other hand, Nimalaratne et al. [59], after investigating the potential transfer of polyphenols from cereals (corn and wheat-based diets) to egg yolk, concluded that only trace amounts of ferulic acid were detected. Additionally, these authors pointed out that free aromatic amino acids, such as tryptophan and tyrosine, were the major contributors to the total antioxidant capacity of egg yolk extracts. Our results (Figure 2 and Figure 3) showed the presence of gallic acid in egg yolk from laying hens fed both control and GP diets. This could be explained by the fact that gallate esters are commonly used as antioxidants in both food and animal feed. Gallate compounds possess antioxidant capacity in both aqueous and lipid media, scavenging peroxy radicals [61]. Interestingly, we also observed that egg yolks from hens fed the GP diet presented a gallic acid content higher than that of eggs obtained from hens fed the control diet, suggesting the potential transfer of some dietary grape polyphenols to the eggs. Previous works indicated a relatively high bioavailability of gallic acid, as proved by the presence in different tissues of its metabolites (like 3-O-methylgallic, 4-O-methylgallic, and 3-4-O-dymethylgallic), together with the parent compounds, after 1–2 h of ingestion [62]. However, in the present study, we have only detected the presence of gallic acid in the egg yolk and none of these metabolites. To the best of our knowledge, the presence of gallic acid in eggs had not been previously reported. An increased transfer of gallic acid to the eggs could contribute to improving their antioxidant capacity. As a consequence, the antioxidant capacity in both aqueous and lipid media might protect egg white and egg yolk alike and could be associated with the effect observed in HU as well as in yolk color, which were both improved in hens fed the diet containing GP. As aforementioned, a high HU value is an indicator of albumen quality and egg freshness. It has been demonstrated that the incorporation of different vegetable sources in hen diets can be used as a nutritional strategy to increase eggs’ self-life and improve the preservation of their nutritional value [63].

## 4. Conclusions

The inclusion of grape pomace in the diet of laying hens enabled an enrichment of egg yolks in gallic acid and an enhancement of some egg quality parameters like albumen Haugh units and yolk color score. However, it also resulted in thinner eggshells, although these changes were not reflected in mechanical parameters. Despite the diet containing grape pomace being richer in unsaturated fat and hence more prone to oxidation of yolk lipids, it did not lead to decreased concentrations of α- and γ-tocopherol in the yolks. The influence of hen genetics was confirmed in parameters such as egg weight, shell thickness, and vitamin concentration in the yolk. Further research on the use of grape by-products in the feed of laying hens should tackle some aspects still scarcely evaluated, like sensory qualities and consumer acceptance of eggs laid by hens fed diets including grape by-products, and should also endeavor to accomplish a more exhaustive identification of the phenolic compounds present in these eggs.

## Figures and Tables

**Table 1:** Proximate composition (g/100 g) of grape pomace.

Nutrients	Grape Pomace Composition
Ether extract	7.5
Crude protein	10.4
Crude fiber	25.3
Ashes	5.1
Total extractable polyphenols (g gallic acid equivalents/100 g DM)	2.10

**Table 2:** Phenolic profile (mg/g DM) of grape pomace.

Compound	Retention Time (min)	Mass	mg/g
Gallic acid	5.2	(−)169,0149	0.07
Gallocatechin	6.7	(+)307,1648	0.06
Delphinidin-3-glucoside	7.9	(+)465,1005	0.25
Cyanidin-3-glucoside	9.6	(+)449,1099	0.16
Petunidin-3-glucoside	10.7	(+)479,1169	0.66
Procyanidin B1	11.4	(−)577,1367	0.16
Procyanidin B2	14.3	(−)577,1367	0.15
Procyanidin B3	9.9	(−)577,1367	0.16
Catechin	12.4	(−)289,0733	0.19
Peonidin-3-glucoside	12.7	(+)463,1217	0.25
Malvidin-3-glucoside	13.2	(+)493,1326	1.61
Epicatechin	15.9	(−)289,0731	0.12
Procyanidin E3	17.1	(−)865,1985	0.18
Procyanidin E4	18.1	(−)1153,2619	0.01
Vitisin A	18.3	(+)561,1217	0.05
Myricetin-3-glucoside	19.8	(−)479,0848	0.03
Malvidin-3-acetylglucoside	20.5	(+)535,1433	0.25
Delphinidin 3-(6-coumaroylglucoside)	20.8	(+)611,1399	0.01
Rutin (quercetin-3-rutinoside)	22.3	(−)609,1331	0.01
Procyanidin B5	22.9	(−)577,1367	0.03
Epicatechin-3-gallate	23.2	(−)441,0866	0.04
Malvidin 3-caffeoylglucoside	23.3	(+)655,1655	0.19
Quercetin-3-galactoside	23.4	(−)463,0892	0.06
Quercetin-3-glucoside	23.8	(−)463,0892	0.07
Cyanidin 3-(6-coumaroylglucoside)	24.1	(+)595,1440	0.03
Quercetin-3-glucuronide	24.2	(−)477,0684	0.17
Peonidin 3-(6-coumaroylglucoside)	25.2	(+)609,1597	0.02
Malvidin 3-(6-coumaroylglucoside)	26.8	(+)639,1705	0.36
Kaempherol-3-glucoside	27.1	(−)447,0942	0.02
Myricetin	30.6	(−)317,0303	0.03
Quercetin	38.4	(−)301,0340	0.09
Kaempherol	43.9	(−)285,0442	0.01

**Table 3:** Ingredient and nutrient composition of experimental diets (g/kg as fed).

Ingredients	Experimental Diets	
	Control	GP ^1^ 50
Corn	500.8	468.6
Soybean	295.0	293.2
Sunflower oil	54.0	63.1
Grape pomace	-	50.0
Straw	25.0	-
Salt	3.0	3.0
Monocalcium phosphate	12.5	12.5
Calcium carbonate	92.5	92.5
Vitamin–mineral premix ^2^	5.0	5.0
DL-Methionine	2.2	2.1
Celite ^3^	10.0	10.0
Analyzed composition		
Crude protein	166	169
Ether extract	76.0	86.0
Crude fiber	35.0	35.0
Total extractable polyphenols (g gallic acid equivalents/kg)	0.644	0.728
Calculated composition		
Grape extractable polyphenols (g gallic acid equivalents/kg) ^4^	-	1.05
AME ^5^ (MJ/kg)	11.4	11.4
Calcium	38.6	38.9
Available P	3.70	3.70
Lysine	8.85	8.85
Meth+Cys	7.86	7.74

^1^ GP = Grape pomace. ^2^ Vitamin–mineral mix supplied the following per kilogram of diet: vitamin A, 12,320 IU; vitamin D_3_, 4620 IU; vitamin E, 15.4 IU; vitamin K, 3.08 mg; riboflavin, 6.16 mg; niacin, 46.2 mg; vitamin B_12_, 23.1 μg; pantothenic acid, 15.4 mg; folic acid, 0.31 mg; choline, 401 mg; Fe, as FeSO_4_, 50.4 mg; Zn, as ZnO, 71 mg; Mn, as MnO, 90 mg; Cu, as CuSO_4_, 7 mg; I, as ethylenediamine dihydroiodide, 0.7 mg; and Se, as Na_2_SeO_3_, 0.25 mg. ^3^ Celite Corp, Lompoc, CA, USA. ^4^ Calculated on the basis of the analyses of polyphenol concentration in GP. ^5^ AME = apparent metabolizable energy.

**Table 4:** Effect of laying hen genetics and dietary inclusion of grape pomace (GP) on the productive performance of 24- to 28-week-old laying hens.

Genetics	ISA White		ISA Brown		SEM ^1^	*p*		
Diet	Control	GP 50	Control	GP 50		Genetics	Diet	Interaction
Daily egg production (%)	97.2	95.7	97.6	98.1	0.839	0.10	0.50	0.20
Average egg weight (g)	57.0	57.3	59.4	59.1	0.691	0.002	0.95	0.65
Daily egg mass (g/d)	55.5	54.9	58.0	58.0	0.975	0.004	0.71	0.73
Feed intake (g/d)	111	111	124	123	1.41	<0.001	0.81	0.82
Feed conversion ratio (g feed/g egg mass)	2.01	2.03	2.14	2.13	0.047	0.012	0.90	0.75

^1^ *n* = 3 replicates (5 hens per replicate).

**Table 5:** Effect of laying hen genetics and dietary inclusion of grape pomace (GP) on quality parameters in eggs of 24- to 28-week-old laying hens.

Genetics	ISA White		ISA Brown		SEM ^1^	*p*		
Diet	Control	GP 50	Control	GP 50		Genetics	Diet	Interaction
Shell (%)	10.6	10.4	10.1	10.2	0.137	0.017	0.97	0.24
Shell thickness (μm)	368	373	380	392	6.0	0.038	0.21	0.56
Haugh units	93.8	96.3	98.6	99.0	1.30	0.003	0.23	0.38
Egg yolk color								
Lightness, L*	43.5	45.6	43.5	44.5	1.06	0.61	0.15	0.58
Redness, a*	3.35	2.51	3.72	2.95	0.14	0.008	<0.001	0.82
Yellowness, b*	27.2	25.1	27.8	26.7	0.89	0.23	0.07	0.59

^1^ *n* = 3 replicates (6 eggs per replicate).

**Table 6:** Effect of egg storage time and dietary inclusion of grape pomace (GP) on Haugh units and yolk color score in eggs of 31- to 34-week-old ISA white laying hens.

Storage Time	0 Days		15 Days		21 Days		31 Days		SEM ^1^	*p*		
Diet	Control	GP 50	Control	GP 50	Control	GP 50	Control	GP 50		Storage Time	Diet	Interaction
Haugh units	95.1	98.4	93.3	93.6	85.1	89.8	69.9	69.3	0.70	<0.001	0.06	0.22
Yolk color score	9.62	10.4	9.56	10.5	9.29	10.7	9.59	10.2	0.217	0.41	<0.001	0.27

^1^ *n* = 3 replicates (9 eggs per replicate).

**Table 7:** Effect of dietary inclusion of grape pomace (GP) on eggshell quality in eggs of 31- to 34-week-old ISA white laying hens.

Diet	Control	GP 50	SEM ^1^	*p*
Average egg weight (g)	63.5	63.2	0.29	0.68
Shell thickness (μm)	424	361	8.6	<0.001
Shell-breaking strength (N)	46.4	45.6	1.50	0.55
Total rupture area (N.s)	441	413	15.5	0.21
Shell rupture force peaks	92.6	82.6	3.84	0.06

^1^ *n* = 3 replicates (18 eggs per replicate for egg weight and shell thickness; 9 eggs per replicate for shell-breaking strength, total rupture area, and shell rupture force peaks).

**Table 8:** Effect of laying hen genetics and dietary inclusion of grape pomace (GP) on α- and γ-tocopherol and retinol content (μg/g) in the yolk of eggs from 24- to 28-week-old laying hens.

Genetics	ISA White		ISA Brown		SEM ^1^	*p*		
Diet	Control	GP 50	Control	GP 50		Genetics	Diet	Interaction
α-tocopherol	87.7	104	139	142	14.8	0.005	0.55	0.65
γ-tocopherol	35.3	41.0	22.1	22.5	5.03	0.004	0.56	0.60
retinol	42.1	42.0	57.2	47.5	6.64	0.14	0.44	0.48

^1^ *n* = 3 replicates (8 eggs per replicate).

## Data Availability

The data presented in this study are available on request from the corresponding author. They are not publicly available because of privacy restrictions.
